# Establishing *Halomonas* as a chassis for industrial biotechnology: advances in synthetic biology tool development and metabolic engineering strategies

**DOI:** 10.1186/s12934-025-02757-2

**Published:** 2025-06-12

**Authors:** André A. B. Coimbra, Satya Prakash, José I. Jiménez, Leonardo Rios-Solis

**Affiliations:** 1 Department of Biochemical Engineering, Bernard Katz Building, University College London, Malet Pl, London, WC1E 6BT UK; 2 Department of Life Sciences, Imperial College London, South Kensington, London, SW7 2AZ UK; 3 Imperial Centre for Engineering Biology, Imperial College London, London, SW7 2AZ UK

**Keywords:** Halomonas, Industrial biotechnology, Non-sterile bioprocessing, Synthetic biology, Metabolic engineering, Bioplastic, Ectoine, chassis

## Abstract

**Background:**

*Halomonas* species have recently emerged as promising chassis organisms for next-generation industrial biotechnology, due to their ability to thrive under high-salt conditions, where most microorganisms cannot survive. This feature minimizes contamination risks, thus enabling cultivation under open, unsterile conditions. In addition, many *Halomonas* species naturally produce large amounts of the bioplastic polyhydroxybutyrate and the high-value osmolyte ectoine.

**Main text:**

This review explores the development of genetic manipulation tools and their pivotal role in establishing the genus *Halomonas* as an industrial chassis. Key additions to the synthetic biology toolbox, including cloning vectors, genetic parts, and genome editing systems are highlighted, along with challenges faced for their adoption, such as difficulties in transformation. In addition, we showcase how these tools have been employed for the development of more robust, high-producing strains through metabolic engineering, as well as for expanding the portfolio of target metabolites produced by *Halomonas*.

**Conclusion:**

Recent developments in synthetic biology tools and metabolic engineering highlighted in this review underscore the potential of *Halomonas* for large scale metabolite production and provide a promising outlook towards their role as a microbial chassis in industrial biotechnology.

## Background

Many chemicals, food additives, and other essential goods have been traditionally sourced from fossil fuel derivatives, carrying a high carbon footprint associated with their production [1, 2]. Industrial biotechnology has thence emerged as a solution to the increasing demands for sustainable and environmentally friendly production of such materials, to gradually replace the petrol-based industry [3, 4]. Currently, industrial biotechnology relies on well-characterized chassis, with the ability to grow to high cell densities and produce large amounts of target metabolites. These chassis include the bacteria *Escherichia coli, Bacillus subtilis, Corynebacterium glutamicum,* and *Cupriavidus necator,* as well as yeasts such as *Saccharomyces cerevisiae* and *Yarrowia lipolytica* [5–10].

However, certain long-standing challenges have limited the competitiveness of current industrial biotechnology (CIB) compared to synthetic chemical processes or natural sources, including high consumption of fresh water, complex and expensive downstream processing for target chemical purification, and contamination risks [11, 12]. Indeed, rigorous sterilization procedures are required to minimize such risks. These procedures can be quite elaborate, energy-intense and expensive, requiring costly stainless-steel bioreactors, and involving high-temperature or high-pressure treatment of both the fermenter vessels and piping systems [13]. Additionally, such sterilization requirements often limit the fermentation type to discontinuous processes, such as batch and fed-batch fermentations, which negatively affects productivity [3].

**Fig. 1:**
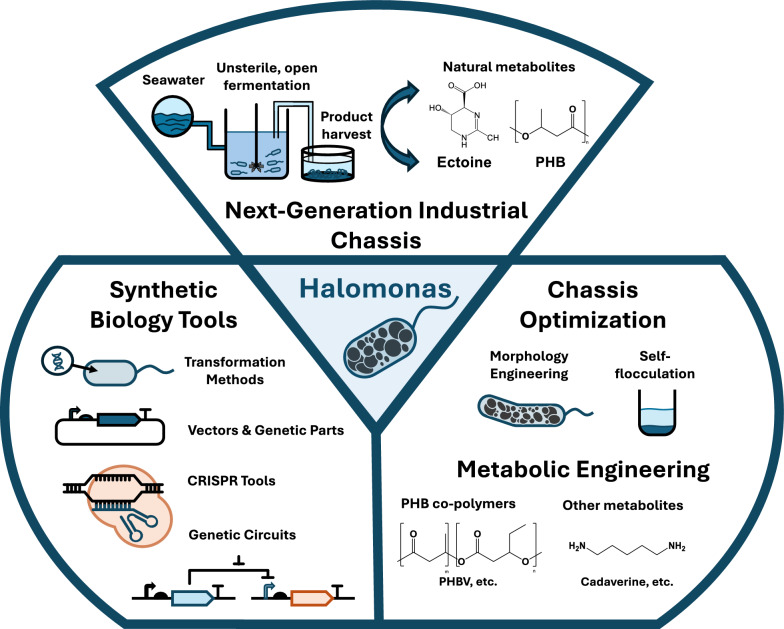
Overview of *Halomonas* as a chassis for industrial biotechnology. *Halomonas* can thrive under high-salt conditions, thus allowing for cultivation under open, unsterile conditions, with minimal contamination risks. Many *Halomonas* species naturally produce large amounts of the bioplastic polyhydroxybutyrate (PHB) and the high-value osmolyte ectoine. Several synthetic biology tools have been developed for *Halomonas*, including transformation methods, vectors, genetic parts, genetic circuits, and CRISPR-based tools. The development of these methods has allowed for chassis robustness to be improved, as well as for different target metabolites to be produced, including different PHB copolymers

To address these issues, a next-generation industrial biotechnology (NGIB) has been developed based on the use of robust organisms resistant to contamination. NGIB employs organisms that thrive in extreme conditions, in which most conventional microorganisms cannot survive, thus eliminating the need for sterilization procedures, and reducing production costs [3]. For example, Moustogianni et al. reported that non-sterile fermentation reduced lipid production costs by a factor of 5 in a working fermentation volume of 24,000 L [14]. Such extremophile organisms include acidophiles (pH < 3), alkaliphiles (pH > 10), halophiles (3–30% NaCl w/v), and thermophiles (temperature: > 50 ºC) [15]. In particular, species of the genus *Halomonas* have become very attractive candidate hosts for microbial cell factory engineering due to their ability to grow well under both high salt and high pH conditions, as well as for their capacity to metabolize a wide range of substrates as carbon sources [4, 16, 17]. In this review, we introduce the *Halomonas* genus as promising chassis for NGIB, highlighting recent advances in the development of genetic manipulation tools and their application in the metabolic engineering of *Halomonas* (Fig. 1).

## Halomonas species as NGIB chassis

*Halomonas* are Gram-negative bacteria commonly found in high-saline environments, such as salt lakes, marshes, and oceans, among others [18]. They are commonly divided into two subtypes according to the salt concentration that provides optimal growth: moderate (3–15% NaCl w/v) and extreme (> 20% NaCl w/v) halophiles [4]. The high-salt tolerance exhibited by *Halomonas* is mainly controlled by two different regulatory mechanisms: the accumulation of inorganic ions (e.g. K^+^) to balance the osmotic pressure caused by the extracellular NaCl [19]; and the production of soluble osmolytes, including ectoine, hydroxyectoine, betaine, and several amino acids, such as glycine, valine and proline, which form an intracellular barrier that prevents external NaCl influx [20]. In recent years, the execution of -omics studies has been instrumental in further elucidating the mechanisms underlying osmoregulation in *Halomonas*. Transcriptome and proteome analyses of *Halomonas elongata* under different NaCl concentrations have revealed upregulation of genes involved in flagellar assembly, chemotaxis, ectoine metabolism, and ABC transporters in high salt conditions [21–23]. In contrast, genes involved in the tricarboxylic acid cycle and fatty acid metabolism are downregulated. Notably, Yu et al. also observed that the expression of sugar transporters was upregulated at 13% NaCl, suggesting enhanced uptake of osmoprotectants under high osmotic stress [21].

On top of their halophilic properties, *Halomonas* can survive under a wide range of temperatures up to 50 ºC, and in alkaline environments with pH over 10 [12]. These characteristics allow for *Halomonas* to be cultured under open unsterile and continuous conditions using wastewater or seawater [12, 24]. Additionally, since sterilization is not required, they can be cultured and maintained in low-cost bioreactors made from cement or plastic, which could be particularly attractive for low-income communities. To date, the genomes of over 500 *Halomonas* strains have been sequenced and deposited in NCBI [25]. Among these, several strains of high industrial potential have been identified, such as *Halomonas boliviensis* [26–28], *H. elongata* [21, 29],* Halomonas* sp. KM-1 [30–32], and *Halomonas smyrnensis* AAD6 [33–35]. In particular, *Halomonas bluephagenesis* [24] has thus far been the focus of most of the research carried out in *Halomonas* spp., with great efforts dedicated to the development of genetic manipulation tools to establish this species as a robust and versatile industrial chassis.

### Natural products

The natural ability of most *Halomonas* species to accumulate large amounts of the biodegradable polyester polyhydroxybutyrate (PHB) have made them one of the most promising microorganisms for bioplastic production (Table 1). In particular, wild-type *H. boliviensis* LC1, *H. venusta* KT832796, and *H. bluephagenesis* TD01 have been shown to accumulate particularly high PHB contents and titers (Table 1) [24, 28, 36]. In fact, *H. bluephagenesis* TD01 has been developed as a low-cost and high yield chassis for PHB production under unsterile and continuous conditions in seawater, reaching a titer of 64.74 g/L and a productivity of 1.46 g/L/h in a 6 L bioreactor [24]. Other species such as *H. campaniensis* LS21 [37], *H. halmophila* 18H [38], *H. halophila* [39], and *H. smyrnensis* AAD6 [34] have shown more moderate PHB accumulation (Table 1). However, the studies for the latter three species were performed under shake flask conditions, which typically produce lower performance metrics.

**Table 1:** Polyhydroxybutyrate (PHB) production levels from glucose by different *Halomonas* wild-type strains

Species	Titer (g/L)	PHB content (% CDW)	Productivity (g/L/h)	Scale	References
*H. boliviensis* LC1	35.4	81.0	1.10	2-L Bioreactor; Fed-batch	[28]
*H. bluephagenesis* TD01	64.74*	78	1.46	6-L Bioreactor; Fed-batch	[24]
*H. campaniensis* LS21	7.24	30	0.15*	7.5-L Bioreactor; Fed-batch	[37]
*H. halmophila* 18H	0.76*	63.72	0.01*	Flask	[38]
*H. halophila*	4.59	72.35	0.06*	Shake flask (250 mL)	[39]
*H. smyrnensis* AAD6	1.34	45.8	0.01*	Shake flask (250 mL)	[34]
*H. venusta* KT832796	33.4	88.12	0.32*	2-L Bioreactor; Fed-batch	[36]

^*^Values inferred from available data

In addition to PHB, several *Halomonas* species have been observed to produce high quantities of the osmoprotectants ectoine and hydroxyectoine (Table 2) [21, 40–44]. These are considered high-value compounds, widely used in the cosmetic industry due to their protective activity for proteins and cells [45]. Remarkably, wild-type *H. elongata* DSM2581 has been observed to naturally produce 12.91 g/L of ectoine under 8% NaCl in a 5 L bioreactor, with a productivity of 1.13 g/L/h (Table 2) [21]. Moreover, ectoine titers of 13.96 g/L were obtained in *H. salina* BCRC17875, although with a lower productivity of 0.29 g/L/h (Table 2) [44]. Meanwhile, *H. boliviensis* LC1 has been able to accumulate 6 g/L of ectoine and 8 g/L of hydroxyectoine following 9 h cultivation at 18.5% NaCl [46]. Interestingly, while *H. bluephagenesis* has demonstrated the highest PHB titers [24], it produced only 0.63 g/L ectoine when grown in 500 mL shake flasks (Table 2) [40].

**Table 2:** Ectoine production levels from glucose by different *Halomonas* wild-type strains

Species	Titer (g/L)	Productivity (g/L/h)	Scale	References
*H. bluephagenesis* TD1.0**	0.63	0.02*	Shake flask (500 mL)	[40]
*H. boliviensis* LC1	5.7	0.14	2-L Bioreactor; Fed-batch	[41]
*H. cupida* J9U**	3.06	0.05	Shake flask (500 mL)	[42]
*H. elongata* DSM2581	12.91	1.13	5-L Bioreactor; Batch	[21]
*H. hydrothermalis* Y2	1.91	0.06*	Shake flask (500 mL)	[43]
*H. salina* BCRC17875	13.96	0.29	Shake flask (250 mL)	[44]

^*^Values inferred from available data

^**^Strains with modifications not aimed at improving metabolite production

Other natural products have also been observed to accumulate in certain *Halomonas* species, such as levan, an extracellular polysaccharide (EPS) composed of fructose monomers widely used in the food industry as an emulsifying, stabilizing and thickening agent [4, 47, 48]. *Halomonas* also constitute a source of industrially relevant enzymes, due to their resistance to organic co-solvents and high pH, temperatures, and salt concentrations [49]. Several enzymes of high industrial potential from *H. elongata* DSM2581 have been identified, including L-asparaginases for chemotherapeutic treatments [50], esterases for the synthesis of anti-inflammatory drugs [51], and a DABA transaminase and an acetyltransferase for their use in the pharmaceutical and cosmetic industries [52, 53], among others [49]. In particular, the novel ω-transaminase HEWT promises to be particularly useful for pharmaceutical synthesis, food chemistry, and polymer synthesis, given its broad substrate scope, active range of amino acid donors and acceptors, and cosolvent tolerance [54].

### Broad-substrate metabolism

Another factor that makes *Halomonas* attractive as an industrial chassis is their ability to utilize different substrates for producing target metabolites. For example, *H. boliviensis* has shown high yields of PHB production when grown on glucose, sucrose, sodium acetate and/or butyric acid [26–28]. Likewise, *H. halophila* was demonstrated to produce PHB from a variety of different sugars, including glucose, fructose, sucrose, cellobiose, xylose, mannose, galactose, rhamnose, and arabinose [55].

*Halomonas* spp. have also shown great potential in utilizing waste materials as a substrate for PHB production, further cementing their position as a sustainable alternative to chemical-based production (Table 3). For instance, *H. alkalicola* has been reported to produce PHB using fruit peel hydrolysates as the sole carbon source, yielding up to 0.45 g/L PHB in 50 mL shake flasks [56]. Meanwhile, *H. cerina* YK44 produced PHB yields of 79.7% CDW from a combination of molasses and soybean flour [57] (Table 3). *H. boliviensis*, in particular, has demonstrated a remarkable capacity to metabolize a wide variety of waste sources, efficiently producing PHB from hydrolysates of wheat bran [58], quinoa stalk [59], oil palm empty fruit bunch and gluten [60], cereal mash [61], and seaweed waste [62] (Table 3). Likewise, Kucera et al. showed the ability of *H. halophila* to utilize several different complex substrates, such as cheese whey, molasses, and hydrolysates from spent coffee grounds, sawdust and corn stover, as a carbon source for the production of PHB, achieving PHB contents between 38.32% and 64.06%, depending on the substrate [55] (Table 3). Later, Kourilova et al. [39] further demonstrated that *H. halophila* could also be used for efficient PHB production from lignocellulosic hydrolysates, including soft-wood, hardwood, rice straw, sugar can bagasse, wheat straw and wheat bran, using synthetic mimic solutions [39] (Table 3). Notably, soft-wood and wheat straw model hydrolysates rendered higher yields (Y_P/S_ [g/g]: 0.28) compared to the glucose control (Y_P/S_ [g/g]: 0.27).

**Table 3:** Polyhydroxybutyrate production levels from different waste sources by different *Halomonas* strains

Species	Waste	PHB titer (g/L)	PHB content (% CDW)	Scale	References
*H. alkalicola*	Banana peel hydrolysates	0.39	16.53	Shake flask (50 mL)	[56]
	Mango peel hydrolysates	0.394	16.60	Shake flask (50 mL)	[56]
	Orange peel hydrolysates	0.45	16.92	Shake flask (50 mL)	[56]
	Pineapple peel hydrolysates	0.28	14.58	Shake flask (50 mL)	[56]
*H. boliviensis* LC1	Cereal mash	26	51.6	2L-Bioreactor; Fed-batch	[61]
	Oil palm empty fruit bunch + gluten hydrolysates	2.3	35.7	Shake flasks (250 mL)	[60]
	Red seaweed waste hydrolysates	3.4	41.1	Shake flasks (500 mL)	[62]
	Residual quinoa stalk hydrolysates	0.34*	24.6*	Shake flasks (250 mL)	[59]
	Wheat bran hydrolysates	4	50	2L-Bioreactor; Batch	[58]
*H. cerina* YK44	Sugarcane molasses + Soybean flour	7.3	79.7	5 mL Culture	[57]
*H. halophila*	Cheese whey hydrolysates	3.26	38.32	Shake flasks (250 mL)	[55]
	Corn stover hydrolysates (2X diluted)	0.82	38.67	Shake flasks (250 mL)	[55]
	Hardwood model hydrolysate	1.96	51.30	Shake flasks (250 mL)	[39]
	Molasses	2.57	64.06	Shake flasks (250 mL)	[55]
	Rice straw model hydrolysates	1.81	51.43	Shake flasks (250 mL)	[39]
	Sawdust hydrolysates	1.00	46.85	Shake flasks (250 mL)	[55]
	Soft-wood model hydrolysates	4.75	73.48	Shake flasks (250 mL)	[39]
	Spent coffee grounds hydrolysates	2.17	61.95	Shake flasks (250 mL)	[55]
	Sugar cane bagasse model hydrolysates	1.74	56.14	Shake flasks (250 mL)	[39]
	Wheat bran model hydrolysates	1.14	44.47	Shake flasks (250 mL)	[39]
	Wheat straw model hydrolysates	3.98	74.74	Shake flasks (250 mL)	[39]
*H.* sp. YLGW01	Red seaweed waste hydrolysates	3.88	62	Test tube (25 mL)	[63]

^*^Values inferred from available data

An important consideration when using waste-derived substrates is the potential presence of microbial inhibitors, which can adversely affect microbial growth and metabolism. Kourilova et al. observed that *H. halophila* growth was completely inhibited by ferulic acid (1 g/L) and significantly inhibited by levulinic acid (1 g/L), which are commonly present in lignocellulose hydrolysates [39]. Removal of such compounds is therefore essential for improving metabolite production. In fact, Bhatia et al. demonstrated that the removal of toxic phenolic compounds, such as furfural and hydroxymethylfurfural, using biomass-derived biochar, improved PHB production from macroalgal (red seaweed) biomass hydrolysates in *Halomonas* sp. YLGW01 by 50% [63].

## Genetic manipulation

The development and standardisation of genetic manipulation tools is essential for improving the cost-efficiency of bioproduction, as well as for extending the portfolio of target metabolites through metabolic engineering [64]. Thus, in the last few years, efforts have focused on the development of such tools for *Halomonas* species, including cloning vectors, expression tuning systems, and CRISPR/Cas technologies. However, this progress has encountered several challenges, primarily due to difficulties in transforming *Halomonas* and issues related to origin of replication incompatibilities.

### DNA mobilisation

One of the main challenges that the genetic manipulation of *Halomonas* has faced is the difficulty in developing efficient transformation protocols. While electroporation is one of the most commonly used transformation methods in bacterial genome engineering, the sensitivity of *Halomonas* towards the non-ionic buffers used in electroporation, along with their dense cell membrane and extensive surface-loaded exopolysaccharides have severely limited its use [4, 65, 66]. Similarly, their dense cell membrane and their broad temperature tolerance have also greatly restricted the use of chemical transformation procedures, leaving conjugation as the most widely used method.

Given the aforementioned limitations of electroporation and chemical transformation, most studies have resorted to RP4-mediated conjugation for the transformation of expression vectors into *Halomonas* [67]. This method was first used in 1995 by Vargas et al., who mobilized the vector pHS15 from *E. coli* DH5α to *H. elongata, H. subglaciescola, H. israelensis*, and* H. halodurans*, using the helper plasmid pRK2013 [68]. However, conjugation is restricted to conjugative or mobilizable plasmids and presents several other limitations, such as DNA fragment size, and the inability to introduce linear DNA fragments into the recipient cell [66]. Additionally, conjugation is relatively low-throughput, restricting the use of high-throughput-dependent methods, required for large dataset mining and analysis [4].

The development of efficient electroporation protocols has proved challenging for *Halomonas* spp. As recently as 2024, Jung et al. reported that they were unable to electroporate *H. shengliensis*, *H. sulfidoxydans*, and *H. elongata*, one of the most well-studied *Halomonas* species [69]. Nevertheless, a few relatively successful attempts have been reported in recent years. In 2016, Harris et al. became the first group to report an electroporation protocol for a *Halomonas* species [70]. By growing the cells in a lower ionic strength medium followed by washes with sucrose (300 mM) prior to electroporation, transformation of *Halomonas* sp. O-1 yielded 10^4^ transformants per μg of DNA. However, this method showed limited reproducibility in other species [66, 71]. In *H. bluephagenesis*, so far only an EPS mutant with improved membrane permeability has been successfully electroporated, with an efficiency of only 400 transformants per µg of DNA [66]. More recently, Park et al. [72] successfully electroporated *Halomonas* sp. YLGW01 by pre-treating the cells with CaCl_2_ (50 mM) prior to sucrose (200 mM) washes [72]. Later that year, Jung et al. electroporated *Halomonas* sp. YK44 with an efficiency of approximately 10^3^ transformants per μg of DNA, although the protocol required an extensive 36 h recovery period following electroporation [69]. Despite these advancements, the efficiency levels of these protocols still remain significantly lower than those of bacteria such as *E. coli* and *P. aeruginosa*, where transformants are obtained in the order of 10^7^–10^9^ per μg DNA [73, 74].

Other methods for transforming *Halomonas* have also sporadically been reported. Seaman and Day discovered and characterized the large temperate phage φgspC, which allowed for transfer of rifampicin resistance between *H. salina GSP21* at frequencies of 4.9 × 10^–8^ and 1.2 × 10^–7^ transductants per recipient, from a multiplicity of infection (MOI) of 0.1 and 0.01, respectively [75]. Moreover, they were also able to transfect *H. venusta* GSP4 with an efficiency of 6.2 × 10^–6^ transductants per recipient at an MOI of 0.1. Meanwhile, Shu et al. chemically transformed *H. campaniensis* XH26 by treating the cells with pre-cooled MgCl_2_ and polyethylene glycol [76]. Still, these cases represent only an exception to the ubiquitous use of conjugation for transformation procedures.

### Plasmid vectors

The development of genetic manipulation tools has also been hindered by the limited compatibility of plasmid origins of replication between different *Halomonas* spp. For example, while the broad-host origins of replication RK2 and pRO1600/ColE1 are compatible with *H. bluephagenesis* [77], *Halomonas sp.* YK44 was not able to maintain plasmids carrying these origins of replication [69]. To address this issue, efforts have been made towards the discovery and development of expression vectors suitable for *Halomonas*. For instance, in 2022, Tsuji, Takei and Azuma developed two vectors, pUCpHAw and pHA1AT_32, based on the *ori* regions from two plasmids isolated from *Halomonas sp.* A020 [71]. Nevertheless, plasmids from the Standard European Vector Architecture (pSEVA) have been the vectors of choice for most of the genetic manipulation studies conducted in *Halomonas*, due to their stability and high conjugative capability [78–80]. Importantly, many plasmids available at SEVA possess broad-host range origins of replication (RK2, pBBR1, etc.), which facilitates genetic work with non-model organisms such as *Halomonas*. On the other hand, antibiotic selection in *Halomonas* can be influenced by salt concentration. The susceptibility of various halophiles, including *H. elongata*, to different antimicrobials has been shown to vary depending on salinity, as shown for the aminoglycosides gentamicin, kanamycin, and streptomycin [81]. The toxicity of these antibiotics in halophiles tends to increase at low salt concentrations, while others, such as rifampicin, remain largely unaffected.

Meanwhile, to facilitate plasmid curing, the temperature-sensitive plasmid pTKmf (Ori pSC101) that replicates at 30 ºC but is destabilized at 37 ºC has also been developed and used effectively in *H. campaniensis* LS21 [37]. Moreover, Ren et al. developed the recombinant plasmid vector pHbPBC based on the novel hcpC/hbpC toxin-antitoxin system found in the endogenous *H. bluephagenesis* plasmid [82]. This toxin-antitoxin system ensures stable maintenance of the plasmid, thus eliminating the need for antibiotics or other selection pressures.

### Genetic parts

Over the years, a series of genetic elements have been developed for gene expression regulation in *Halomonas*, including promoters, ribosome binding sites (RBSs) and terminators.

#### Promoters and gene expression tuning systems

**Fig. 2:**
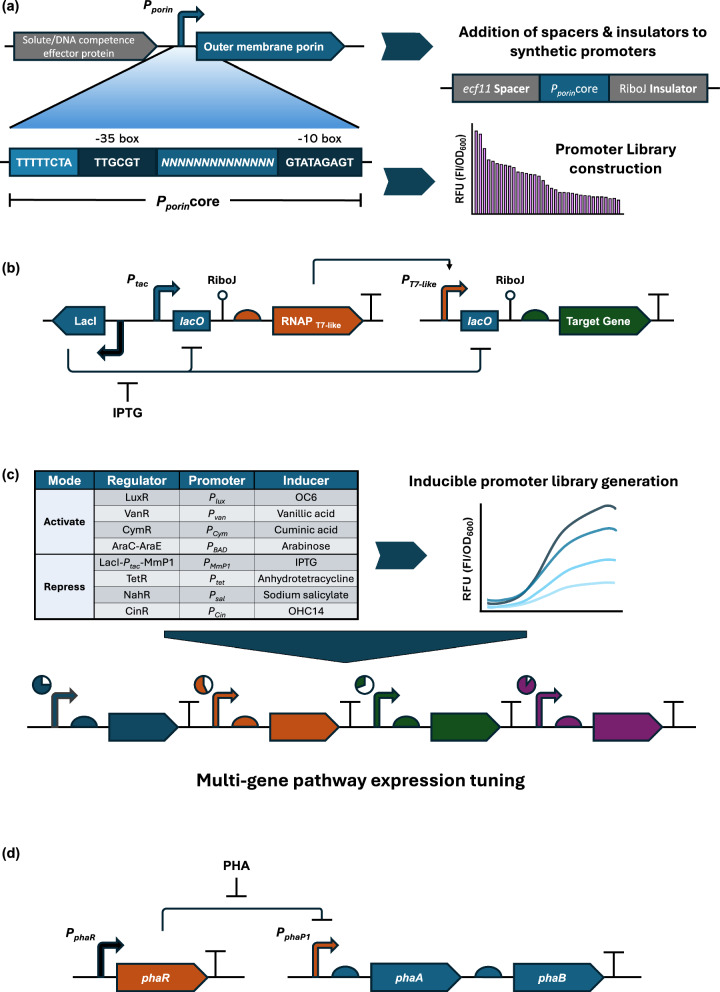
Development of promoters and expression tuning systems in *Halomonas.* **a** Construction of promoter libraries based on the promoter of an outer membrane porin (*P*_*porin*_). Promoter libraries were generated by mutagenesis of the promoter region between the − 35 and − 10 boxes within the *P*_*porin*_core. Control of expression levels was enhanced by flanking *P*_*porin*_core with an *ecf11* spacer upstream and a RiboJ insulator downstream [83, 84]. **b** Development of T7-like expression systems. The expression of the target gene is under the control of a T7-like RNA polymerase (RNAP_T7-like_). In turn, expression of the RNAP_T7-like_ is regulated by LacI, which also represses the expression of the target gene. Addition of IPTG, which binds the *lac* repressor, activates expression of the RNAP_T7-like_ and, consequently of the target gene [90]. **c** Development of expression tuning systems. Different inducible promoter systems have been generated for *Halomonas*. Inducible promoter libraries with different dynamic ranges were then developed from these systems. The availability of multiple orthogonal inducible systems allows for multi-gene pathway expression tuning [93]. **d** Development of a self-stimulating system. Overexpression of the PHA-production genes *phaA* and *phaB* is regulated by the repressor PhaR. In turn, PhaR is inhibited by PHA accumulation. Hence, PHA accumulation stimulates further PHA production [95]

Given that *E. coli* promoters behave unpredictably in *Halomonas* spp., due to the unique genetic background and transcription machinery of these species, Li et al. decided to generate a promoter library based on the promoter of a highly expressed porin protein from *H. bluephagenesis* TD01 that they identified and characterized [83]. By randomizing the sequence between the − 35 and − 10 elements, they successfully constructed a constitutive promoter library with a 310-fold variation in transcriptional activity (Fig. 2a). Moreover, by integrating a *lac* operator into the core region of the porin promoter (P_*porin*_), they were also able to generate an inducible promoter library with a > 200-fold induction. Trisrivirat et al. later also designed and screened a constitutive promoter library by random mutagenesis of the same porin promoter [11]. Shen et al. further complemented the *Halomonas* promoter toolbox by devising a constitutive promoter in which the P_*porin*_ promoter was flanked upstream by the spacer sequence from the *ecf11* promoter derived from *Pseudomonas syringae* and downstream by the RiboJ insulator [84–87]. These features conferred enhanced stability to the engineered promoter and allowed for the construction of a promoter library with a relative transcriptional strength ranging from 40 to 140,000 that was amenable to use both in *H. bluephagenesis* and *E. coli* (R^2^ = 0.98) [84].

To fine-tune the expression of metabolic pathways, it is key to develop inducible systems. The T7 expression system, derived from the phage T7 RNA polymerase gene, is one of the most popular systems for recombinant protein production in *E. coli* [88, 89]. However, since this system was not viable in *H. bluephagenesis*, Zhao et al. developed three T7-like RNA polymerase-promoter pairs (MmP1, VP4, and K1F9) through phage genome database mining, followed by in vivo characterisation (Fig. 2b) [90]. These phage-derived expression systems displayed orthogonality, tight regulation, and high efficiency. Other ligand-based inducible systems have also been developed and tested. For instance, a synthetic FadR-based oleic acid (OA)-induced system has been constructed in *H. bluephagenesis* with a dynamic control range of 150-fold and reduced expression leakage [91]. In another study, Ma et al. used a quorum sensing-based regulator LuxR, which, in the presence of acyl homoserine lactone (AHL), triggers the transcription of the corresponding promoter P_*lux*_. [40]. Inducible systems have also already been developed and tested in other *Halomonas* spp. For instance, Park et al. have used the inducible promoter LacI^q^-P_*trc*_ to tune the expression of *atoAD* in *H*a*lomonas* JJY01 [72].

Furthermore, the simultaneous use of multiple induction systems has enabled the development of more complex expression tuning systems, allowing for enhanced metabolite production in *H. bluephagenesis*. For instance, in 2020, Ma et al. combined the LuxR-AHL and T7-like inducible systems to fine-tune the transcription levels of three clusters related to ectoine synthesis [40]. In a later study, Wang et al. employed the inducible promoters P_*lac*_ and P_*lux*_, induced by IPTG and AHL, respectively, to fine-tune the expression levels of *phaC*_*ac*_* and phaJ*_*ac*_, resulting in enhanced production of poly(3-hydroxybutyrate-co-3-hydroxyhexanoate) (PHBHHx) [92]. Recently, Ma et al. (2024) constructed and characterized ten inducible promoters and their respective translational regulators: AraC-Ara, CinR-OHC14, CymR-Cuma, NahR-Sal, VanR-Van, TetR-aTC, LuxR-OC6, TtgR-Nar, PhlF-DAPG, LacI-MmP1 RNAP-IPTG (Fig. 2c) [93]. Subsequently, libraries of inducible promoters were developed for each of the expression systems, with dynamic ranges varying from 10^2^ to 7 × 10^4^ FI (a.u.). Moreover, the authors showed that, given their orthogonality, several expression systems could be used at the same time to modulate metabolite production. In this case, LacI-MmP1 RNAP-IPTG, LuxR-OC6, CinR-OHC14 and AraC-Ara were used to fine tune the expression of lycopene biosynthesis pathway in *H. bluephagenesis*, resulting in a 6-fold improvement in yield.

Since the addition of inducers in large-scale fermentations is not cost-effective, once optimal expression levels have been identified using inducible systems, they can be replaced by constitutive promoters with equivalent expression level [94]. In *H. bluephagenesis*, this process has been successfully employed by Ma et al., who effectively substituted the inducible PHB-production construct pP_*MmP1*_-*phaC*-P_*Cin_GCCC*_-*phaA*-P_*Cym*_-*phaB* with a constitutive one pP_*Porin68*_-*phaC*-P_*Porin221*_-*phaA*-P_*Porin199*_-*phaB*, without compromising PHB production [93]. Meanwhile, a different strategy was adopted by Zheng et al., as they developed a self-stimulating system that did not rely on external inducers (Fig. 2d) [95]. The system is based on a P_phaP1_* promoter, controlled by the transcriptional regulator PhaR, which is inhibited at high PHB concentrations. This self-stimulating mechanism allowed for a 13.5% increase in PHB content in *H. bluephagenesis*.

#### Other genetic parts

Besides promoters, other genetic elements are also important for regulating transcription. Genetic insulators are valuable tools that allow promoter characterization to be standardized across genetic constructs [87]. To this effect, Ma et al. developed a library derived from the insulator RiboJ that produced an expression level ranging from 10^3^ to 10^5^ relative fluorescence units [93]. Likewise, transcription terminators play a key role in preventing unwanted RNA polymerase read-through [96]. Additionally, they can contribute to mRNA stabilization and enhancing protein expression [97–100]. Accordingly, a series of intrinsic Rho-independent terminators have been developed for *H. bluephagenesis* by both genome mining and rational design [101]. Seven terminators exhibited higher efficiencies than the canonical strong T7 terminator (75.3%), of which three terminators displayed efficiencies above 90%. Meanwhile, the initiation of mRNA translation is largely regulated by its ribosome binding site (RBS). Thus, RBS libraries can effectively be used for tuning metabolic pathways [102, 103]. To this end, Ma et al. recently developed an RBS library with transcriptional activity ranging from 10^0^ to 10^5^ relative fluorescence units [93]. Overall, the development of these libraries has provided a versatile toolbox for fine-tuning gene expression, which will prove useful for metabolic pathway engineering optimization, as well as other synthetic biology applications.

### Genome editing tools

While plasmid vectors constitute a useful platform for the expression of recombinant genes, they can give rise to unexpected metabolic burden, resulting in reduced growth and product synthesis [104]. Additionally, in an industrial setting, plasmids can be hard to maintain for long durations and at larger scales, resulting in large variability in copy numbers and production efficiencies [93]. Moreover, plasmid maintenance relies on the use of antibiotics or selection markers, thus making the production process more expensive. Chromosomal integration of genetic constructs can address this issue, providing a more stable and reliable alternative for recombinant gene expression.

#### Suicide plasmid-mediated editing

Homologous recombination-based genome editing tools allow for precise deletions and integration of genetic constructs. In *Halomonas* species, this approach was first employed in 2005 by Kraegeloh et al., who introduced the suicide vector pK18mobsacB in *H. elongata* to delete the *teaABC* operon [105]. Later, Fu et al. also used a suicide vector to produce gene knock-outs in *H. bluephagenesis* TD01 by markerless gene replacement [106]. However, due to the labour-intensive and time-consuming nature of recombination-based gene editing, recent studies have shifted focus towards different alternatives.

#### CRISPR/Cas9-based genomic editing

CRISPR/Cas9-based technologies have been popularized as the method of choice for genomic insertions and deletions [83, 107–109]. In *H. bluephagenesis*, Qin et al. developed a CRISPR/Cas9 system using a dual-plasmid approach, comprised of a low copy number plasmid pSEVA321 that carried the *Streptococcus pyogenes* Cas9 gene under the control of its native promoter, and a high copy number plasmid pSEVA241 equipped with both the sgRNA and the donor DNA flanked by 500-bp homology arms [110]. Using this system, the authors were able to delete different target genes with varying efficiencies, ranging from 12.5% for *phaB* to 100% for *zwf*. Additionally, they demonstrated that this system could also be applied in other *Halomonas* species, as they conducted targeted deletion of the *minCD* genes in *H. campaniensis* LS21 with an efficiency of 96%.

In addition to gene knock-outs, Qin et al. also employed this system to perform targeted gene insertion in *H. bluephagenesis* [110]. However, low efficiency was observed for inserting fragments of lengths above 3 kb, likely due to the bacterium’s poor ability to repair double-strand breaks by homology-directed repair (HDR). Incidentally, the authors attempted to incorporate the λ-red recombination system into *H. bluephagenesis*, which has been demonstrated to enhance HDR efficiency in other prokaryotes [110, 111]. However, this system was not functional in *H. bluephagenesis*, significantly reducing editing efficiency. Hence, to enable the insertion of larger genetic fragments, the homology arms of the insertions were extended to 1 kb. Using this strategy, Qin et al. successfully knocked-in the genes *4hbD*, *sucD* and *ilbB* (~ 4.5 kb) in order to produce 4-hydroxybutyrate [110].

Meanwhile, in 2022, Xu et al. employed a different strategy to address the limitations of HDR in *H. bluephagenesis* by constructing a dual-sgRNA system [66]. Using this system, they were able to efficiently delete larger genomic fragments, such as non-essential gene clusters, including the flagella, exopolysaccharides and O-antigen. However, a significant decline in editing efficiency was observed with increasing fragment sizes, dropping to 25% for 40 kb and 12.5% for 50 kb. To address these limitations, Liu et al. later used a CRISPR/Cas9-assisted non-homologous end joining (NHEJ) editing system to improve the deletion efficiency of large DNA fragments [112]. By co-expressing the *Ku* and *LigD* genes from the NHEJ system of *Mycobacterium tuberculosis* alongside the CRISPR/Cas9 system, they successfully deleted a 50 kb flagellar cluster targeted by three sgRNAs with an efficiency of 31%. However, while useful for performing deletions of large genomic clusters, NHEJ-CRISPR-based systems are inherently limited by the unprecise and unpredictable nature of the process [113]. This is undesirable for situations where the generation of controlled and reproducible genomic modifications is necessary, such as generating industry-grade bacterial strains.

#### Base editing

Base editors have also become increasingly popular tools for genome editing purposes. The fusion of a dead or nicking Cas9 protein (dCas9 and nCas9, respectively) to an adenosine or cytidine deaminase allows for the generation of single-base pair modifications in the protospacer region [114]. Base editors can target multiple *loci* at once and exhibit a lower rate of off-target modifications when compared to traditional Cas9 systems. Additionally, cytidine base editors can effectively be used to generate functional knock-outs by converting amino acid-encoding codons (arginine, glutamine, and tryptophan) into STOP codons.

In *H. bluephagenesis*, phosphoenolpyruvate carboxykinase (*pck*) knock-outs were produced using the CRPISR/AID system, which comprises of a pSEVA321 plasmid encoding for a dCas9 fused to a cytosine deaminase, and a pSEVA341 plasmid expressing the gRNA [115]. Recently, Zhang et al. (2024) developed a multiple-*loci* genome editing system named CRISPR-deaminase-assisted base editor (CRISPR-BE), comprised of a cytidine (CRISPR-cBE) and an adenosine (CRISPR-aBE) deaminase-based base editors fused to a nCas9 [116]. Using CRISPR-BE, cytidine to thymidine, and adenosine to guanosine mutations were introduced in *H. bluephagenesis* with an efficiency of up to 100% within a 7-nt editing window. Effective CRISPR-cBE multiplexing was further demonstrated by inactivating all six copies of the gene IS1086 in *H. bluephagenesis*.

### Gene expression modulation tools

#### CRISPR interference and small RNA-mediated repression

The development of gene repression tools, such as CRISPR interference (CRISPRi) and small RNAs, has been important for the expansion of the synthetic biology toolbox of *Halomonas*. In 2017, Tao, Lv, and Chen used CRISPRi for the first time in *H. bluephagenesis* to repress the expression of the *ftsZ* gene, leading to the formation of elongated cells [117]. In addition, CRISPRi has been used to fine tune gene expression in metabolic pathway engineering. In the previous study, CRISPRi was also employed to regulate the expressions of the *prpC* gene, encoding 2-methylcitrate synthase, for regulating the 3-hydroxyvalerate monomer ratio in poly(3-hydroxybutyrate-co-3-hydroxyvalerate) (PHBV).

Importantly, gene repression tools allow for the modulation of the expression of essential genes that cannot be knocked-out by conventional CRISPR/Cas9 tools. In *H. bluephagenesis*, repression of the essential gene *gltA*, encoding for citrate synthase, using CRISPRi allowed more acetyl-CoA to be channelled from the tricarboxylic cycle to PHB synthesis [117]. Beyond this, these tools have also been employed to determine the function of certain genes and their influence on certain metabolic pathways. For instance, in 2022, Wang et al. determined the influence of 30 genes on mevalonate production in *H. bluephagenesis* using a small RNA library generated using the PrrF1-2-HfqPa system, a highly efficient gene expression regulation system developed for *H. bluephagenesis* [118].

## Metabolic engineering of *Halomonas* species

### Chassis optimization

Besides the aforementioned exploitation of the natural production of PHB and ectoine displayed by *Halomonas* species, further optimization of metabolite output is necessary for NGIB to compete with the chemical industry. Metabolic engineering thence plays a key role in enabling the construction of high-producing strains. For example, Ma et al. employed a multiple inducible system to enhance PHB content by 15% and cell-dry weight (CDW) by 30% in *H. bluephagenesis* [93]. Meanwhile, Zheng et al. improved CDW, PHB content (%) and productivity by 20.4, 13.5, and 36.4%, respectively, by employing PHA self-stimulating system [95]. Using this system, they obtained titers of 74.32 g/L PHB in a 7 L bioreactor in a fed-batch culture of *H. bluephagenesis* ZS19 (Table 4). These production values show that *Halomonas* is capable of outperforming other organisms that naturally produce PHB, such as *C. necator* (25.7 g/L; 51% CDW; 0.43 g/L/h) [119], as well as other traditional chassis organisms that have been engineered to produce PHB, such as *E. coli* (36.3 g/L; 66.5% CDW; 1.2 g/L/h) [120].

**Table 4:** Metabolite production levels from engineered *Halomonas bluephagenesis*

Metabolite	Titer (g/L)	Yield	Productivity (g/L/h)	Scale	Sterilization	References
2-Pyrrolidone	6.3	0.21 (g/g GABA)*	0.13*	Shake flask	–	[126]
3-(3.4-Dihydroxyphenyl)-L-alanine	0.974	–	–	–	Sterile	[127]
3-Hydroxypropionate	154	0.93 (g/g 1.3-propanediol)	2.4	7-L Bioreactor; Fed-batch	Open fermentation	[128]
5-Aminovaleric acid	67.4	–	0.77*	7-L Bioreactor; Fed-batch	Open fermentation	[129]
5-Hydroxyvalerate	6	0.3 (g/g Lysine)*	0.25	Shake flask (100 mL)	Sterile	[129]
Acetoin	85.84	0.46 (g/g pyruvate)*	10.73*	Bottle (25 mL); Fed-batch	Sterile	[130]
Ectoine	84.6	0.29 (g/g glucose)	1.63	7-L Bioreactor; Fed-batch	Open fermentation	[121]
Gamma-Aminobutyric acid	357.2	–	–	7-L Bioreactor; Fed-batch	Open fermentation	[126]
Itaconic Acid	63.60	0.63 (g/g citrate)	1.12	Shale Flask (150 mL)	Sterile	[131]
L-Threonine	33	0.07 (g/g glucose)*	1.40*	7-L Bioreactor; Fed-batch	Open fermentation	[132]
Lycopene	0.01086	–	–	Deep-well Plate	Sterile	[93]
Mandelate	0.071	–	–	450-mL PhotoBioreactor; Semi-continuous fed-batch	Open fermentation	[133]
Mevalonate	121	0.42 (g/g glucose + acetic acid)	1.01*	5-L Bioreactor; Fed-batch	Open fermentation	[134]
Poly(3-hydroxybutyrate-co-3-hydroxyhexanoate)	31.62*	–	0.658*	7-L Bioreactor; Fed-batch	Open fermentation	[92]
Poly(3-hydroxybutyrate-co-3-hydroxyvalerate)	4.1*	–	0.085*	Shake Flask	Sterile	[135]
Poly(3-hydroxybutyrate-co-4-hydroxybutyrate	79.5	–	1.59	7-L Bioreactor; Fed-batch	Open fermentation	[84]
Poly(3-hydroxybutyrate-co-5-hydroxyvalerate)	2.94*	–	0.06*	Shake Flask	Sterile	[129]
Polyhydroxybutyrate	74.32*	–	2.06	7-L Bioreactor; Fed-batch	Open fermentation	[95]
Propane	–	116.3 (mg/g CDW)	–	450-mL PhotoBioreactor; Semi-continuous fed-batch	Open fermentation	[133]

^*^Values inferred from available data

In *H. elongata*, ectoine biosynthesis has been improved by overexpressing glucose transporters [29], while Shu et al. removed competing pathways in *H. campaniensis* XH26 [76]. Recently, Hu et al. (2024) developed an ectoine hyperproducing strain of *H. bluephagenesis* by overexpressing the *ectABC* operon, increasing precursor availability, enhancing the product transport system and optimizing the growth medium [121]. The resulting strain produced 85 g/L ectoine in 52 h in a 7 L bioreactor under open unsterile conditions (Table 4). While this titer is still lower than the highest reported ectoine titer obtained from engineered *E. coli* (131.8 g/L) [122], the productivity and yield on glucose were slightly higher (*H. bluephagenesis*: 1.63 g/L/h, 0.29 g/g glucose; *E. coli*: 1.16 g/L/h, 0.26 g/g glucose), thus showing that *H. bluephagenesis* can be a competitive chassis for ectoine production.

The ability to produce metabolites from different sugar sources is crucial for establishing an organism as an industrial chassis. Hence, expanding the range of substrates available for metabolite production through metabolic engineering is a key focus in strain development. To this end, Liu et al. introduced the gene encoding for the α-amylase Amy03713, which allowed *H. bluephagenesis* to produce 6.32 g/L of PHB from starch in shake flasks [123].

Chassis performance can also be further improved by engineering the morphology and physiology of the cell. For example, Qin et al. expressed the *Vitreoscilla* hemoglobin (VHb) gene to improve the oxygen supply in *H. bluephagenesis* TD01, resulting in improved growth [110]. Meanwhile, Tao, Lv and Chen repressed the expression of *ftsZ*, a key gene in bacterial fission, producing larger cells with an up to 70-fold increase in their length [117]. Deletion of non-essential genes can also reduce the metabolic burden and improve the stability of the host cell [124]. In *H. bluephagenesis*, Xu et al. deleted approximately 3% of the genome, including genes encoding for flagella, exopolysaccharides and O-antigen [66]. The authors also observed that engineered exopolysaccharides mutants rapidly self-flocculated and precipitated within 20 min. without centrifugation, thus facilitating downstream processing.

A major challenge limiting the broader application of bio-production is strain instability in large-scale bioreactors. This issue affects various host microbes, including *E. coli*, *C. glutamicum*, and *H. bluephagenesis*, often leading to failure during scale-up. To address this issue, Zhang et al. deleted the 8 main transposases from the genome of *H. bluephagenesis* WZY278 to decrease genomic instability [125]. Indeed, they observed greater consistency and stability in CDW and PHA accumulation over a 48 h growth period in a 7-L bioreactor.

### Production of non-natural metabolites

**Fig. 3:**
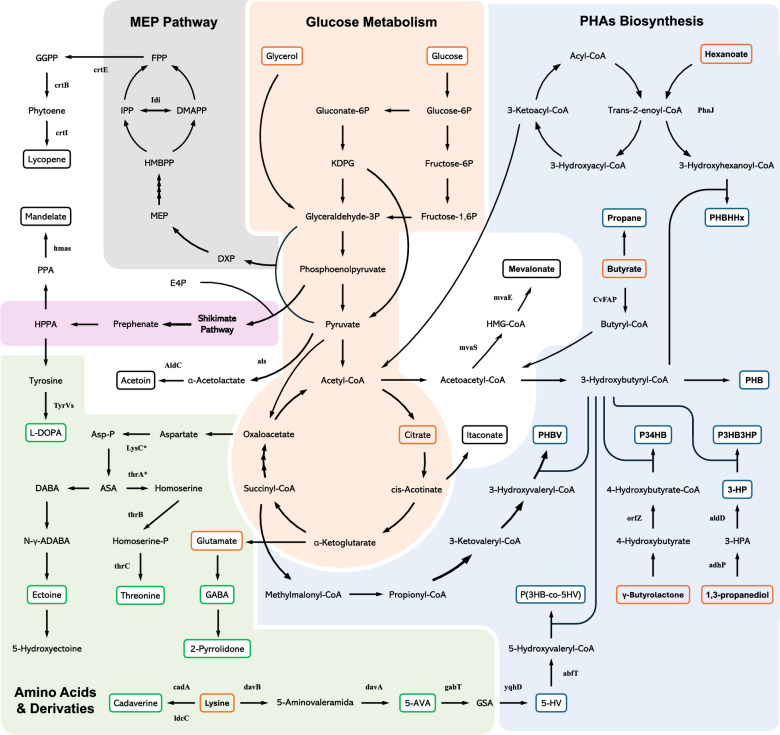
Engineered pathways for metabolite production in *Halomonas*. *Halomonas* metabolic pathways have been divided into different modules: Glucose Metabolism (orange); PHAs Biosynthesis (blue); Amino Acids & Derivatives (green): MEP Pathway (gray); Shikimate Pathway (pink). Compounds used as substrates for metabolite production are circled in orange. Target metabolites are encircled in the colour corresponding to the respective metabolic module. Heterologously expressed enzymes for metabolite production have been annotated

While *Halomonas* species can produce PHB in very large quantities, PHB as a plastic is quite rigid, brittle and highly crystalline, properties which make it unsuitable for most uses [136, 137]. As such, efforts have been focusing on producing PHB co-polymers with better properties (Table 4, Fig. 3). For instance, Wang et al. engineered *H. bluephagenesis* to express a PHA synthase (PhaCac) and an enoyl coenzyme-A hydratase (PhaJac) from *Aeromonas caviae* FA440 to produce PHBHHx [92], which is highly flexible and has similar properties to petroleum-derived plastics [138–140]. In another study, *H. bluephagenesis* was engineered to produce poly(3-hydroxybutyrate-co-4-hydroxybutyrate) (P34HB), with a productivity of 1.59 g/L/h after 50 h of growth under non-sterile fed-batch conditions [84]. Remarkably, production of PHB and its derivatives in *H. bluephagenesis* has been scaled up successfully up to 5000 L, achieving a cell dry weight (CDW) of 100 g/L, of which 60.4% was P34HB following a 36 h non-sterile fermentation [141]. Other PHB co-polymers have also been produced in *H. bluephagenesis*, including poly(3-hydroxybutyrate-co-3-hydroxypropionate) (P3HB3HP), poly(3-hydroxybutyrate-co-5-hydroxyvalerate) [P(3HB-co-5HV)], and PHBV (Table 4, Fig. 3) [128, 129, 135].

*Halomonas* can also be efficiently engineered to produce high levels of other target metabolites (Table 4, Fig. 3). For instance, *H. campaniensis* LC-9 was engineered to produce cadaverine from L-lysine by recombinant expression of the two lysine decarboxylases LdcC and CadA (Fig. 3) [115]. In the presence of 0.05% Triton X-100, used for enhanced cell membrane permeability, the conversion efficiency of L-lysine to cadaverine reached 100%, resulting in a titer of 118 g/L of cadaverine. Meanwhile, *H. bluephagenesis* has been engineered to produce 5-aminovaleric acid (5-AVA) from L-lysine, with a yield of 67.4 g/L in a 7 L bioreactor (Table 4) [129]. Further engineering allowed for production of 6 g/L of 5-hydroxyvalerate (5HV), and P(3HB-co-5HV) as well (Fig. 3). In another study, 5 exogenous genes were expressed in *H. bluephagenesis* to produce 33 g/L of L-threonine in a 7 L bioreactor under open unsterile conditions (Table 4, Fig. 3) [132]. *H. bluephagenesis* has also been engineered to synthesize other target products, including itaconic acid [131], acetoin [130], 3,4-dihydroxyphenyl-L-alanine (L-DOPA) [127], lycopene [93], gamma-aminobutyric acid (GABA) [126], and 2-pyrrolidone [126] (Table 4, Fig. 3). Importantly, *H. bluephagenesis* has been shown to be an ideal chassis for the production of mevalonate (MVA), a crucial building block for the biosynthesis of isoprenoids [134]. By expressing MVA synthetases from *Enterococcus faecalis* and *Lactobacillus casei*, repressing competing pathways, and introducing a non-oxidative glycolysis (NOG) pathway into *H. bluephagenesis*, Zhang et al. were able to produce 121 g/L of MVA with a yield of 0.42 g/g (glucose + acetic acid) in a 5 L bioreactor, the highest titer and yield for MVA reported in a bioreactor for any microorganism to date (Table 4, Fig. 3). Simultaneous production of multiple compounds in *Halomonas* has also been explored. In 2023, Park et al. engineered *H. bluephagenesis* to produce both the biofuel propane (116.3 mg/g CDW), mandelate (71 mg/L) and PHB (69% g/g CDW) in a semi-continuous batch fermentation under open conditions (Table 4, Fig. 3) [133].

## Final remarks & outlook

*Halomonas* species have emerged as a highly promising bacterial chassis for NGIB, largely due to their ability to grow under high salt conditions, which allows for their cultivation under open, unsterile conditions. Natural producers of PHAs and the high-value compound ectoine, *Halomonas* have also been engineered to produce high yields of other non-natural compounds, such as MVA and GABA. The development of synthetic biology tools has been key to the assertion of *Halomonas* as an NGIB chassis. The generation of genetic part libraries, development of expression tuning systems, and establishment of CRISPR/Cas systems have all been important additions to the *Halomonas* synthetic biology toolkit.

It is important to note, however, that the synthetic biology tool development and research conducted so far have largely been performed on *H. bluephagenesis*. Consequently, many other *Halomonas* species of high industrial potential have remained relatively unexplored. To fully harness the potential of *Halomonas* as an NGIB chassis, it is essential to expand research beyond *H. bluephagenesis* and explore the potential of other species. To this end, -omics studies could play an important role in further understanding the metabolism of different *Halomonas* species and identifying strains that are better suited for producing different target compounds or that exhibit more favourable properties for industrial applications. Promising candidates such as *H. halophila* and *H. boliviensis* have shown high levels of PHB production and broad substrate-range metabolism [26–28, 39, 55]. Yet, no studies on their genetic manipulation have been conducted so far. This gap likely stems from a combination of factors, including the lack of standardization of synthetic biology toolkits amongst *Halomonas* spp., as well as the challenges associated with establishing efficient transformation protocols, as previously discussed. Moreover, limitations in chemical transformation and electroporation have also restrained the performance of high-throughput screening experiments and prevented the wider use of genome engineering tools such as ribonucleoproteins or genome editing using linear DNA, thus deterring further work in the genetic manipulation of different *Halomonas* strains with high industrial potential.

The scalability of the synthetic biology tools developed for *Halomonas* is another critical factor in its success as an industrial chassis. Genetic systems developed at a smaller scale often falter during scale-up. For instance, Ma et al. [93] achieved significant improvements in CDW and PHB production when they expressed *phaCAB* under the control of multiple tuning systems in *H. bluephagenesis* using deep-well 96-well plates [93]. However, massive plasmid loss was observed when scaling up the system to shake flasks. Even with the genomic integration of the *phaCAB* module, PHB production was only restored to wild-type levels. In contrast, the self-stimulating system developed by Zheng et al. [95] proved effective in enhancing PHB production in a 7 L bioreactor, highlighting the importance of developing scale-adaptive systems [95].

While recent studies have been performed on the influence of salt conditions on the transcriptome and proteome of *Halomonas* species [21, 22, 125], few studies have focused on the implications of varying salt concentrations in synthetic biology tool development. For instance, existing promoter libraries developed for *Halomonas* have been largely derived from the promoter of an outer membrane porin (OMP) [11, 83, 84]. However, OMP expression has been observed to be influenced by osmolarity in *H. elongata*, as well as other microorganisms [23, 142–144]. Moreover, given the effect of osmolarity on protein levels and protein resource allocation [125], further research should focus on better understanding how the activity of different ribosome binding sites is influenced by varying salinity and, ultimately, how this could affect chassis performance.

Altogether, developments in synthetic biology tools and metabolic engineering have allowed *Halomonas* species to start to establish themselves as a robust chassis for large scale metabolite production. PHB derived from *H. bluephagenesis* has already been successfully deployed for production at large scale. The startup PhaBuilder in China already produces 10,000 tonnes per annum of PHB, P34HB, and other derivatives [145]. Excitingly, last year the construction of a new PHB production site with a capacity of 30,000 tonnes per annum was initiated, evidencing that implementation of *Halomonas*-derived bioplastic at a large scale is technically and economically feasible. Meanwhile, in the UK, the company C3 Biotechnologies Limited has been launched to produce biofuels, such as kerosene, hypersonic fuels, and liquefied petroleum gas, from waste material using *Halomonas* [146]. The setup of these companies provides a promising outlook for the future of *Halomonas* as a key microbial chassis in industrial biotechnology. 

## Data Availability

No datasets were generated or analysed during the current study.

## Abbreviations

5-AVA: 5-Aminovaleric acid
5HV: 5-Hydroxyvalerate
AHL: Acyl homoserine lactone
CDW: Cell-dry weight
CIB: Current industrial biotechnology
CRISPRi: CRISPR interference
EPS: Extracellular polysaccharide
FI (a.u.): Fluorescence intensity in arbitrary units
GABA: Gamma-aminobutyric acid
HDR: Homology-directed repair
L-DOPA: 3,4-Dihydroxyphenyl-L-alanine
MVA: Mevalonate
nCas9: Nicking Cas9
NGIB: Next-generation industrial biotechnology
NHEJ: Non-homologous end joining
NOG: Non-oxidative glycolysis
OA: Oleic acid
OMP: Outer membrane porin
P(3HB-co-5HV): Poly(3-hydroxybutyrate-co-5-hydroxyvalerate)
P3HB3HP: Poly(3-hydroxybutyrate-co-3-hydroxypropionate)
P34HB: Poly(3-hydroxybutyrate-co-4-hydroxybutyrate)
PHA: Polyhydroxyalkanoate
PHB: Polyhydroxybutyrate
PHBHHx: Poly(3-hydroxybutyrate-co-3-hydroxyhexanoate)
RBS: Ribosome binding site
